# Effects of novel synthetic microneurotrophins in diabetic retinopathy

**DOI:** 10.1186/2193-1801-4-S1-L25

**Published:** 2015-06-12

**Authors:** Silvia Lisa, Ruth Iban-Arias, Despina Kokona, Ioannis Charalampopoulos, Achilleas Gravanis, Kyriaky Thermos

**Affiliations:** University of Crete, Greece

**Keywords:** Diabetic retinopathy, Microneurotrophins, neuroprotection

Diabetic retinopathy (DR) is a neovascular, inflammatory and neurodegenerative disease. The neovascular component is treatable but no therapeutic agents are available for the other two components. Early changes in the diabetic retina include neuronal death of amacrine and retinal ganglion cells (RGC)(Barber et al., J Clin Invest,1998), and elevated levels of inflammatory mediators (Yoshimura et al., Plos One,2009). Nerve Growth factor (NGF) receptors, namely TrkA and p75NTR, are expressed in RGC. The TrkA receptor activates prosurvival, while p75NTR activates inflammatory and apoptotic pathways (Mysona et al., Expert Rev Ophthalmol,2014). Dehydroepiandrosterone (DHEA) binds to both receptors, mimicking NGF. It affords anti-apoptotic, neuroprotective and anti-inflammatory effects in the retina (Kokona et al., Neuro-pharmacol, 2012, Straub et al., J Clin Endocrinol, 1998). The therapeutic use of DHEA is restricted due to its metabolic products. The main objective of this study was to investigate and compare the neuroprotective and anti-inflammatory effects of DHEA and its spiro-epoxy derivatives BNN27 and BNN20(Calogeropoulou et al., J Med Chem,2009) (not metabolized to estrogens and androgens), in the rat streptozotocin model of DR. BNN27, via TrkA activation, protected in a dose-dependent manner (2, 10, 50mg/kg, ip) bNOS (brain nitric oxide synthetase) and TH (tyrosine hydroxylase) expressing amacrine cells and ganglion axons (NFL immunoreactivity) similar to DHEA’s actions, while BNN20 was less effective. BNN27 activated the TrkA prosurvival signaling pathway ERK1/2 kinase. It reduced the activation of SAPK/JNK kinase and the expression of p75NTR. BNN27 also increased the expression of anti-inflammatory cytokines (IL10). These results suggest that NGF TrkA receptor is involved in the neuroprotective and anti-inflammatory effects of BNN27 and is a valuable target via which BNN27 could afford efficacious therapeutics for the treatment of DR.

